# Deep learning reconstruction for lumbar spine MRI acceleration: a prospective study

**DOI:** 10.1186/s41747-024-00470-0

**Published:** 2024-06-21

**Authors:** Hui Tang, Ming Hong, Lu Yu, Yang Song, Mengqiu Cao, Lei Xiang, Yan Zhou, Shiteng Suo

**Affiliations:** 1 Department of Radiology, Renji Hospital, School of Medicine, Shanghai Jiao Tong University, No. 160, Pujian Road, Pudong New District, Shanghai, 200127 China; 2 MR Research Collaboration Team, Siemens Healthineers Ltd., Shanghai, China; 3 Subtle Medical, Shanghai, China; 4 Biomedical Instrument Institute, School of Biomedical Engineering, Shanghai Jiao Tong University, Shanghai, China

**Keywords:** Artificial intelligence, Deep learning, Low back pain, Lumbar vertebrae, Magnetic resonance imaging

## Abstract

**Background:**

We compared magnetic resonance imaging (MRI) turbo spin-echo images reconstructed using a deep learning technique (TSE-DL) with standard turbo spin-echo (TSE-SD) images of the lumbar spine regarding image quality and detection performance of common degenerative pathologies.

**Methods:**

This prospective, single-center study included 31 patients (15 males and 16 females; aged 51 ± 16 years (mean ± standard deviation)) who underwent lumbar spine exams with both TSE-SD and TSE-DL acquisitions for degenerative spine diseases. Images were analyzed by two radiologists and assessed for qualitative image quality using a 4-point Likert scale, quantitative signal-to-noise ratio (SNR) of anatomic landmarks, and detection of common pathologies. Paired-sample *t*, Wilcoxon, and McNemar tests, unweighted/linearly weighted Cohen *κ* statistics, and intraclass correlation coefficients were used.

**Results:**

Scan time for TSE-DL and TSE-SD protocols was 2:55 and 5:17 min:s, respectively. The overall image quality was either significantly higher for TSE-DL or not significantly different between TSE-SD and TSE-DL. TSE-DL demonstrated higher SNR and subject noise scores than TSE-SD. For pathology detection, the interreader agreement was substantial to almost perfect for TSE-DL, with *κ* values ranging from 0.61 to 1.00; the interprotocol agreement was almost perfect for both readers, with *κ* values ranging from 0.84 to 1.00. There was no significant difference in the diagnostic confidence or detection rate of common pathologies between the two sequences (*p* ≥ 0.081).

**Conclusions:**

TSE-DL allowed for a 45% reduction in scan time over TSE-SD in lumbar spine MRI without compromising the overall image quality and showed comparable detection performance of common pathologies in the evaluation of degenerative lumbar spine changes.

**Relevance statement:**

Deep learning-reconstructed lumbar spine MRI protocol enabled a 45% reduction in scan time compared with conventional reconstruction, with comparable image quality and detection performance of common degenerative pathologies.

**Key points:**

• Lumbar spine MRI with deep learning reconstruction has broad application prospects.

• Deep learning reconstruction of lumbar spine MRI saved 45% scan time without compromising overall image quality.

• When compared with standard sequences, deep learning reconstruction showed similar detection performance of common degenerative lumbar spine pathologies.

**Graphical Abstract:**

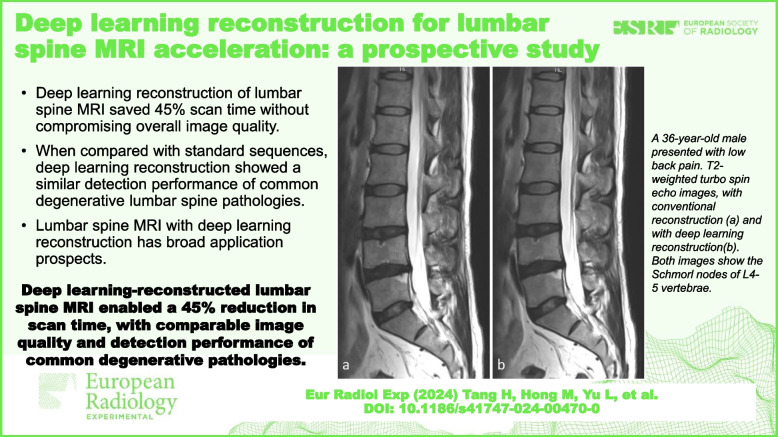

**Supplementary Information:**

The online version contains supplementary material available at 10.1186/s41747-024-00470-0.

## Background

Lumbar pain is a common chronic pain in clinical practice. Various factors contribute to the onset of the disease, and current research indicates that lumbar spine disease is the primary cause of low back pain [1]. Lumbar spine x-ray and computed tomography can show the bony structure of the lumbar spine, but the former does not show the lumbar intervertebral discs and the surrounding soft tissues while the latter shows them with relatively limited contrast resolution, and both of them imply ionizing radiation exposure for the patients. Magnetic resonance imaging (MRI) is widely used for routine examination of lumbar spine diseases due to the absence of ionizing radiation and high soft tissue contrast [2]. However, as lumbar MRI examination takes a relatively long time, patients with severe lumbar spine diseases may experience significant pain during the examination, resulting in body displacement and motion artifacts, which would impede diagnostic accuracy [3]. Research has focused on finding ways to shorten the examination time for the lumbar spine MRI while still meeting the image quality requirements for clinical diagnosis [4–6].

In recent years, with the rapid development of artificial intelligence, deep learning (DL)-based image enhancement techniques have been applied for MRI fast imaging [7–9]. This approach uses neural networks with many layers of processing units to learn complex patterns in large amounts of data [10, 11]. Unlike conventional fast acquisition methods which modify imaging parameters at the cost of reduced clarity and signal-to-noise ratio (SNR), DL-based techniques enable higher-quality reconstruction from undersampled *k*-space data by learning complex mappings between undersampled and fully-sampled data [12]. However, despite its advantages, we are concerned about whether the quality of the images obtained with DL reconstruction is consistent with that of standard scanning protocols and whether the post-processed images truly reflect the nature of abnormities.

In this prospective study, we aimed to compare the image quality and detection performance of common degenerative pathologies in lumbar spine MRI reconstructed using a DL technique with those obtained with standard protocols.

## Methods

### Participants

The study was approved by the Ethics Committee of Shanghai Renji Hospital, China (Ethics No. LY2023-121-B). All subjects signed an informed consent form before the MRI examination. Patients suspected of having degenerative lumbar spine diseases who were scheduled for lumbar spine MRI from October to November 2023 were prospectively enrolled. The exclusion criteria were (1) a history of lumbar spine surgery or implantation of metallic foreign bodies, (2) those who suffered from claustrophobia, (3) those who had contraindications to MRI examination, and (4) those who had intolerable pain in the lumbar region and could not lie down for a long time.

### Imaging protocol

Standard and accelerated lumbar spine MRI examinations were performed on all patients using a Siemens 3-T MRI unit (MAGNETOM Prisma, Siemens Healthcare, Erlangen, Germany). Standard turbo spin-echo (TSE-SD) protocol included sagittal T1-weighted and T2-weighted imaging with no acceleration, transverse T2-weighted imaging with no acceleration, and sagittal fat-suppressed T2-weighted imaging with an acceleration factor of 2 and a number of excitations of 2. Accelerated turbo spin-echo protocol with DL reconstruction (TSE-DL) included the same sequences with an acceleration factor of 2 for non-fat-suppressed imaging and a number of excitations of only 1 for fat-suppressed T2-weighted imaging. The undersampled scans were reconstructed by a dedicated software (SubtleMR V2, Subtle Medical, Menlo Park, USA) using DL algorithms to obtain the processed TSE-DL images. SubtleMR is a US Food and Drug Administration–FDA-cleared and European Conformity–CE-marked software utilizing a deep back-projection network for DL reconstruction of MRI. SubtleMR has been trained and validated on a multicenter dataset of over a million MRI image pairs from various vendors and modalities [13]. It can be utilized for different contrast images including T1-weighted and T2-weighted imaging. Details on the dedicated software and the DL algorithm are summarized in Supplementary Material 1: Appendix 1. The specific parameters of TSE-SD and TSE-DL protocols are detailed in Table 1.

**Table 1:** Technical parameters of standard turbo spin-echo and turbo spin-echo with deep learning reconstruction

	Standard turbo spin-echo				Turbo spin-echo with deep learning reconstruction			
Sequence	Sag-T2	Sag-T2 FS	Sag-T1	Tra-T2	Sag-T2	Sag-T2 FS	Sag-T1	Tra-T2
Thickness (mm)	4	4	4	4	4	4	4	4
TR (ms)	2,400	2,800	621	3,290	2,400	2,800	621	3,290
TE (ms)	92	80	9.2	92	92	80	9.2	92
FOV (mm^2^)	280 × 280	280 × 280	280 × 280	180 × 180	280 × 280	280 × 280	280 × 280	180 × 180
Average	1	2	1	1	1	1	1	1
Matrix	384 × 268	320 × 224	384 × 268	320 × 256	384 × 268	320 × 224	384 × 268	320 × 256
Acceleration factor	None	2	None	None	2	2	2	2
Bandwidth (Hz/Px)	250	252	250	252	250	252	250	252
Scan time (s)	77	70	101	69	41	42	53	39

*FOV* Field of view, *FS* Fat-suppressed, *Sag* Sagittal, *TE* Echo time, *TR* Repetition time, *Tra* Transverse, *T1* T1-weighted, *T2* T2-weighted

### Image analysis

All images were randomly sorted, and image quality and diagnosis evaluation of TSE-SD images and TSE-DL images were performed independently by two radiologists with more than 10 years of experience in lumbar spine imaging. Before performing the actual image analysis, both readers underwent a training session that encompassed datasets not part of the current study. This training was designed to acquaint the readers with the Likert scale classification system detailed in the following paragraph. The readers were blinded to MRI protocol type, clinical information, and radiologic reports. All markers that could potentially identify patients or sequences were removed. To minimize recall bias, interpretations of the TSE-SD and TSE-DL images for each patient were conducted in two sessions separated by 4 weeks. Each reconstruction type and patient order were randomized in the sessions. Randomization was achieved by sorting the datasets using random numbers.

Qualitative image analysis was performed on each series with the use of a 4-point Likert scale for the following items: sharpness of anatomic structures (intervertebral discs, vertebrae, cerebrospinal fluid, intervertebral foramina, spinous processes, small joints, and nerve roots), artifacts, noise, overall image quality, and diagnostic confidence. For sharpness, overall image quality, and diagnostic confidence, the scoring system was as follows: 1, poor; 2, fair; 3, good; and 4, excellent. Artifacts and noise were rated as follows: 1, severe; 2, moderate; 3, mild; and 4, none. Examples of the application of the 4-point Likert scale are shown in Supplemental Fig. S1.

For quantitative image analysis, we placed round or oval regions of interest and measured the SNR on sagittal images for the L1–L5 vertebrae, L1–L5 intervertebral discs, psoas major muscles, cerebrospinal fluid, and fat and on transverse images for the right and left nerve roots, right and left psoas major muscles, L3/4 intervertebral discs, cerebrospinal fluid, and fat. All regions of interest were aligned with the center of the anatomy, while excluding the boundary region. The average size was 204.9 mm^2^ (range 202–206.3 mm^2^) for the vertebrae, 24.7 mm^2^ (21.4–27.6 mm^2^) for the intervertebral discs, 184.8 mm^2^ (182.4–187.2 mm^2^) for the psoas major muscles, 25 mm^2^ (23.4–26.1 mm^2^) for the cerebrospinal fluid, and 29.8 mm^2^ (27.6–30.8 mm^2^) for the fat on sagittal images and 1.1 mm^2^ (1.0–1.3 mm^2^) for the nerve roots, 70.2 mm^2^ (68.4–71.8 mm^2^) for the psoas major muscles, 325.3 mm^2^ (324.1–326.7 mm^2^) for the L3/4 intervertebral discs, 9.2 mm^2^ (6.2–10.8 mm^2^) for the cerebrospinal fluid, and 25.2 mm^2^ (23.4–26.8 mm^2^) for the fat on transverse images. The illustration of the region of interest placement is presented in Supplemental Fig. S2. The following formula was used to calculate the SNR:

$$SNR=\frac{Mean\,signal\,intensity}{Standard\,deviation\,of\,background\,noise}$$

Furthermore, the readers recorded the presence or absence of the following pathologies on a vertebral level: spinal stenosis, foraminal stenosis, intervertebral disc degeneration, disc bulge, disc herniation, facet synovial cyst, Modic changes, and Schmorl nodes.

### Statistical methods

Continuous variables were tested for normality using the Shapiro-Wilk test and reported as means ± standard deviations or medians and interquartile ranges. Categorical variables were reported as numbers and percentages. The paired-sample *t* test or Wilcoxon test were used to determine the differences between the groups. Interreader agreement was assessed by using the unweighted Cohen *κ* statistics for binary variables, linearly weighted Cohen *κ* statistics for ordinal variables, and intraclass correlation coefficients (two-way model, absolute agreement, and single measures) for continuous variables, as well as for the interprotocol agreement. The agreements were interpreted as follows: 0–0.20, poor agreement; 0.21–0.40, fair agreement; 0.41–0.60, moderate agreement; 0.61–0.80, substantial agreement; and 0.81–1.00, almost perfect agreement [14]. The McNemar test was used to compare the differences in detecting major pathologies by TSE-SD and TSE-DL. A two-sided *p* < 0.05 was considered statistically significant. For image quality comparison, the significance level was reduced to an *α*-adjusted *p*-level of 0.05/2 according to Bonferroni correction, where 2 is the number of tests conducted for each aspect of image quality [15]. Statistical analyses were performed using SPSS version 21 (IBM Corp, Armonk, NY, USA).

## Results

### Study participants

Two patients who were unable to adhere to the examination due to back pain were excluded, and finally, a total of 31 patients were enrolled (15 males and 16 females; mean age 51 ± 16 years). All enrolled patients completed the examination with complete TSE-SD and TSE-DL sequences.

### Scan time

The total scan time for TSE-SD and TSE-DL protocols were 317 s (5:17 min:s) and 175 s (2:55 min:s), respectively, with a scan time saving of 45%.

### Image quality

#### Qualitative analysis

**Fig. 1:**
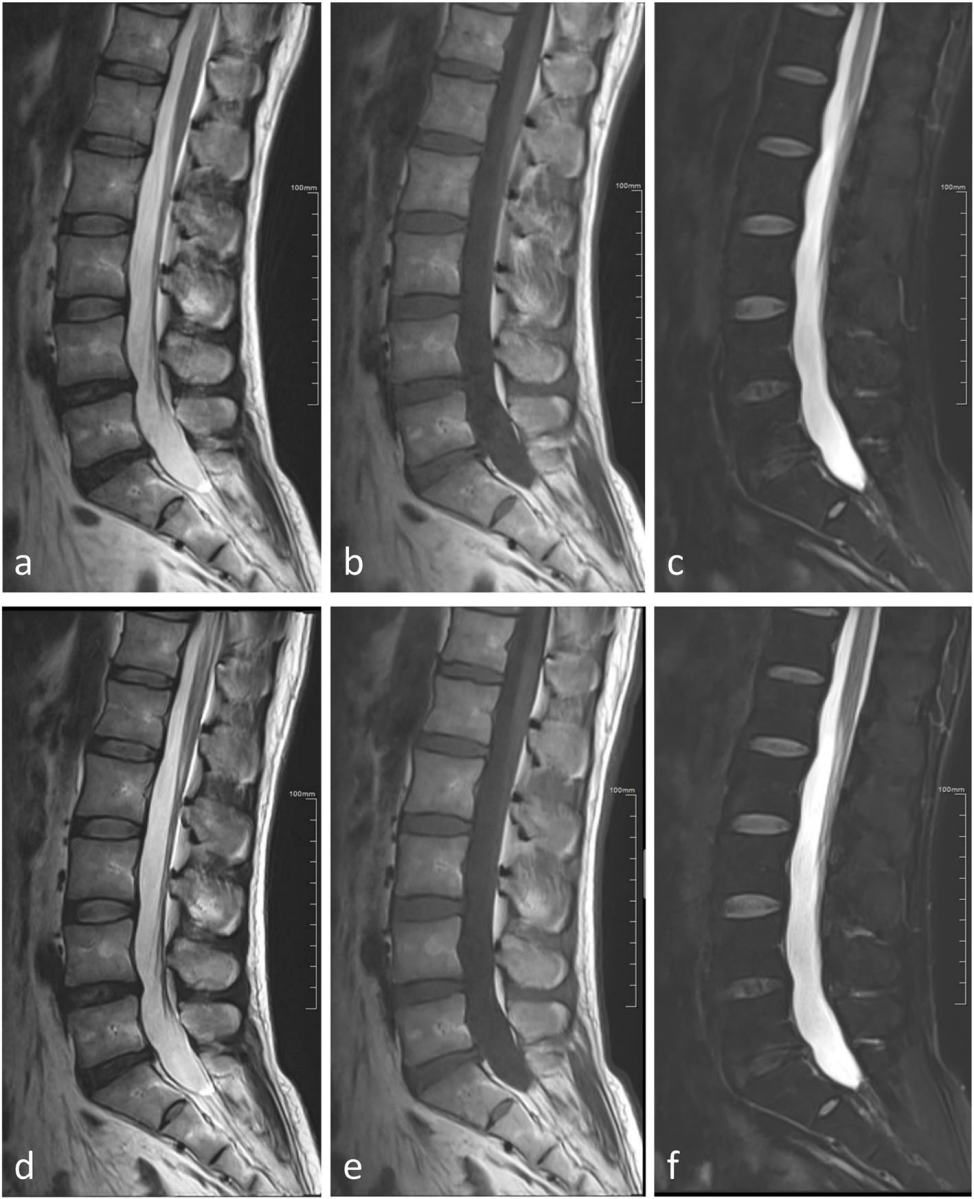
A 38-year-old male with low back pain. Images (**a**, **b**, and **c**) are the sagittal T2-weighted, T1-weighted, and fat-suppressed T2-weighted images obtained with TSE-SD, respectively, while images (**d**, **e**, and **f**) are the sagittal T2-weighted, T1-weighted, and fat-suppressed T2-weighted images obtained with TSE-DL, respectively. TSE-DL images exhibit sharper anatomic structures and decreased noise levels compared with TSE-SD images. TSE-SD, Standard turbo spin-echo; TSE-DL, Turbo spin-echo with deep learning reconstruction

**Fig. 2:**
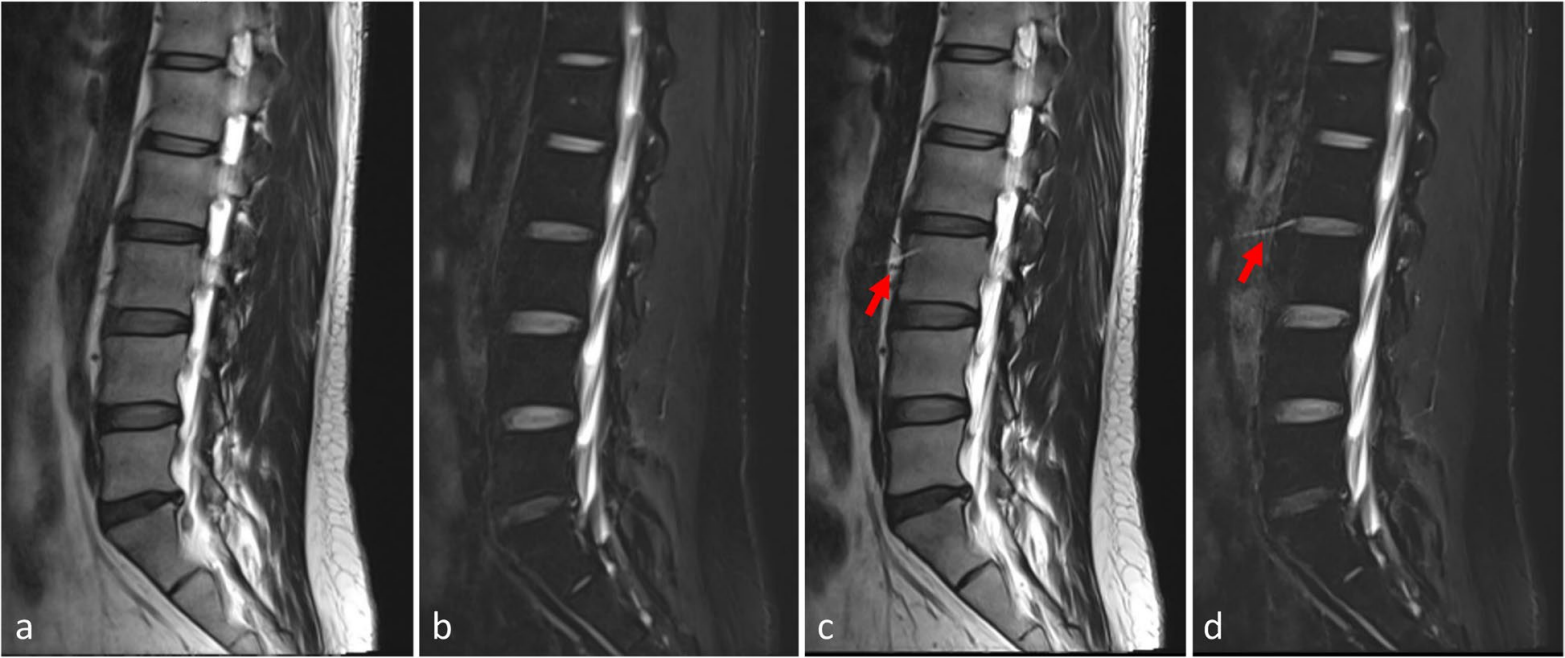
A 40-year-old male presented with left sciatica. Images (**a** and **b)** are sagittal T2-weighted and fat-suppressed T2-weighted images obtained with TSE-SD, respectively, while images (**c** and **d**) are sagittal T2-weighted and fat-suppressed T2-weighted images obtained with TSE-DL, respectively. Residual aliasing artifacts due to undersampling appear on TSE-DL images (arrows). TSE-SD, Standard turbo spin-echo; TSE-DL, Turbo spin-echo with deep learning reconstruction

Results of the qualitative analysis are shown in Table 2 and Supplemental Tables S1, S2 and S3. Reader 1 reported better image sharpness for TSE-DL compared with TSD-SD in non-fat-suppressed sequences (*p* ≤ 0.022) (Fig. 1), whereas reader 2 found no evidence of a significant difference in sharpness (*p* ≥ 0.087). Both readers reported more artifacts on TSE-DL fat-suppressed T2-weighted images than TSD-SD images (*p* < 0.001 for reader 1 and *p* = 0.001 for reader 2) (Fig. 2). No evidence of a significant difference in artifacts was found between TSE-DL and TSE-SD in other non-fat-suppressed sequences (*p* ≥ 0.084). Noise was reduced in TSE-DL compared with TSD-SD in all sequences (Fig. 1), although no significant difference was observed in transverse T2-weighted images for reader 2 after Bonferroni correction (*p* = 0.044). The overall image quality was higher for TSE-DL in non-fat-suppressed sequences, although significant differences were only observed in sagittal T1-weighted images (*p* = 0.003 for reader 1 and *p* = 0.008 for reader 2) and transverse T2-weighted images (*p* = 0.022 for reader 1). In terms of diagnostic confidence, no significant difference was observed between TSE-DL and TSE-SD (*p* ≥ 0.081 for both readers). Interreader agreement for the qualitative image quality analysis was fair to substantial (weighted *κ* = 0.26–0.73), except for the evaluation of artifacts on sagittal T2-weighted images (weighted *κ* = 0.13, poor agreement) (Table 2 and Supplemental Tables S1, S2 and S3).

**Table 2:** Subjective evaluation of sagittal T1-weighted images by two readers

	Reader	TSE-SD	TSE-DL	*p*-value	Cohen *κ*
Sharpness	1	3.61 ± 0.49	4.00	0.011	0.45 (0.28, 0.62)
	2	3.81 ± 0.40	3.90 ± 0.30	0.087	
Artifacts	1	3.90 ± 0.30	4.00	0.084	0.26 (0.05, 0.47)
	2	3.87 ± 0.34	3.84 ± 0.37	0.665	
Noise	1	3.61 ± 0.49	4.00	0.001	0.73 (0.62, 0.84)
	2	3.58 ± 0.50	4.00	< 0.001	
Overall image quality	1	3.71 ± 0.46	4.00	0.003	0.67 (0.54, 0.80)
	2	3.74 ± 0.45	3.97 ± 0.18	0.008	
Diagnostic confidence	1	3.90 ± 0.30	4.00	0.081	0.57 (0.41, 0.74)
	2	3.87 ± 0.34	3.94 ± 0.25	0.161	

Data in parentheses are 95% confidence intervals. Image quality was evaluated with the use of a 4-point Likert scale. For sharpness, overall image quality, and diagnostic confidence, the scoring system was as follows: 1, poor; 2, fair; 3, good; and 4, excellent. For artifacts and noise, the scoring system was as follows: 1, severe; 2, moderate; 3, mild; and 4, none

*TSE-SD* Standard turbo spin-echo, *TSE-DL* Turbo spin-echo with deep learning reconstruction

#### Quantitative analysis

For each anatomy evaluated, TSE-DL images showed better SNR compared with TSE-SD images on each sequence, although reader 2 reported no significant differences in the L1/2 intervertebral disc and fat on sagittal fat-suppressed T2-weighted images after Bonferroni correction (*p* = 0.039 and *p* = 0.048, respectively) (Table 3 and Supplemental Tables S4, S5 and S6). Interreader agreement for SNR measurement of each anatomy was moderate to almost perfect (intraclass correlation coefficient range 0.54–0.94) (Table 3 and Supplemental Tables S4, S5 and S6).

**Table 3:** Signal-to-noise ratio measurement of sagittal T1-weighted images by two readers

	Reader	TSE-SD	TSE-DL	*p*-value	ICC
Vertebra					
L1	1	157.6 ± 39.1	282.5 ± 62.6	< 0.001	0.83 (0.72, 0.90)
	2	149 ± 45	257.4 ± 70.2	< 0.001	
L2	1	154.0 ± 39.9	275.0 ± 66.7	< 0.001	0.85 (0.73, 0.91)
	2	144.5 ± 44.9	248.7 ± 71.6	< 0.001	
L3	1	144.5 ± 35.1	257.8 ± 61.8	< 0.001	0.86 (0.73, 0.92)
	2	135.5 ± 39.4	233.2 ± 67.1	< 0.001	
L4	1	138.3 ± 33.6	247.5 ± 62.0	< 0.001	0.84 (0.68, 0.91)
	2	127.7 ± 35.1	220 ± 62.4	< 0.001	
L5	1	143.3 ± 35.7	254.4 ± 68.7	< 0.001	0.83 (0.71, 0.90)
	2	134.1 ± 38.7	230.3 ± 68.8	< 0.001	
Intervertebral disc					
L1/L2	1	92.9 ± 24.3	169.0 ± 38.8	< 0.001	0.83 (0.73, 0.90)
	2	89.5 ± 26	155.4 ± 42.4	< 0.001	
L2/L3	1	91.5 ± 33.6	164.6 ± 52.5	< 0.001	0.82 (0.72, 0.89)
	2	87.8 ± 27.4	153.5 ± 45.3	< 0.001	
L3/L4	1	82.2 ± 19.2	149.5 ± 34.6	< 0.001	0.85 (0.76, 0.91)
	2	79.2 ± 22.2	137.3 ± 38.8	< 0.001	
L4/L5	1	79.2 ± 22.7	141.8 ± 39.0	< 0.001	0.87 (0.78, 0.92)
	2	75.5 ± 22.8	131 ± 41.4	< 0.001	
L5/S1	1	89.0 ± 24.4	159.8 ± 40.2	< 0.001	0.82 (0.71, 0.89)
	2	84.2 ± 21.6	147.4 ± 41.7	< 0.001	
Muscle	1	128.8 ± 24.7	232.2 ± 38.1	< 0.001	0.75 (0.61, 0.85)
	2	123.1 ± 29.3	213.6 ± 48.2	< 0.001	
Cerebrospinal fluid	1	67.9 ± 17.7	123.2 ± 28.6	< 0.001	0.83 (0.72, 0.89)
	2	64.9 ± 19.1	114 ± 34.2	< 0.001	
Fat	1	366.6 ± 119.9	651.8 ± 196.1	< 0.001	0.74 (0.54, 0.85)
	2	328.1 ± 103.4	561.8 ± 153.8	< 0.001	

Data in parentheses are 95% confidence intervals

*ICC* Intraclass correlation coefficient, *TSE-SD* Standard turbo spin-echo, *TSE-DL* Turbo spin-echo with deep learning reconstruction

### Detection performance of common degenerative pathologies

For detecting common pathologies, the *κ* values of the interprotocol intrareader agreement ranged from 0.84 to 1 for reader 1 and from 0.87 to 1.00 for reader 2, both indicating almost perfect agreement (Table 4). For each protocol, the intraprotocol interreader agreement was moderate to almost perfect for TSE-SD with *κ* values ranging from 0.58 to 1.00 and substantial to almost perfect for TSE-DL with *κ* values ranging from 0.61 to 1.00 (Table 4).

**Table 4:** Intraprotocol interreader and interprotocol intrareader agreement for detecting common abnormalities

	Intraprotocol interreader (TSE-SD)	Intraprotocol interreader (TSE-DL)	Interprotocol intrareader (Reader 1)	Interprotocol intrareader (Reader 2)
Spinal stenosis	0.82 (0.68, 0.96)	0.83 (0.69, 0.96)	0.92 (0.83, 1.00)	0.97 (0.91, 1.00)
Foraminal stenosis	0.58 (0.39, 0.76)	0.64 (0.47, 0.81)	0.86 (0.75, 0.97)	0.94 (0.86, 1.00)
Intervertebral disc degeneration	0.83 (0.74, 0.92)	0.81 (0.71, 0.90)	0.91 (0.84, 0.97)	0.88 (0.81, 0.96)
Disc bulge	0.64 (0.50, 0.77)	0.67 (0.54, 0.81)	0.92 (0.84, 0.99)	0.93 (0.86, 1.00)
Disc herniation	0.86 (0.76, 0.95)	0.81 (0.70, 0.92)	0.95 (0.89, 1.00)	0.93 (0.86, 1.00)
Facet synovial cyst	1.00	1.00	1.00	1.00
Modic changes	0.61 (0.37, 0.84)	0.61 (0.37, 0.84)	0.84 (0.69, 0.99)	0.87 (0.72, 1.00)
Schmorl nodes	0.69 (0.43, 0.95)	0.74 (0.49, 0.98)	1.00	0.94 (0.83, 1.00)

Data in parentheses are 95% confidence intervals

*TSE-SD* Standard turbo spin-echo, *TSE-DL* Turbo spin-echo with deep learning reconstruction

**Fig. 3:**
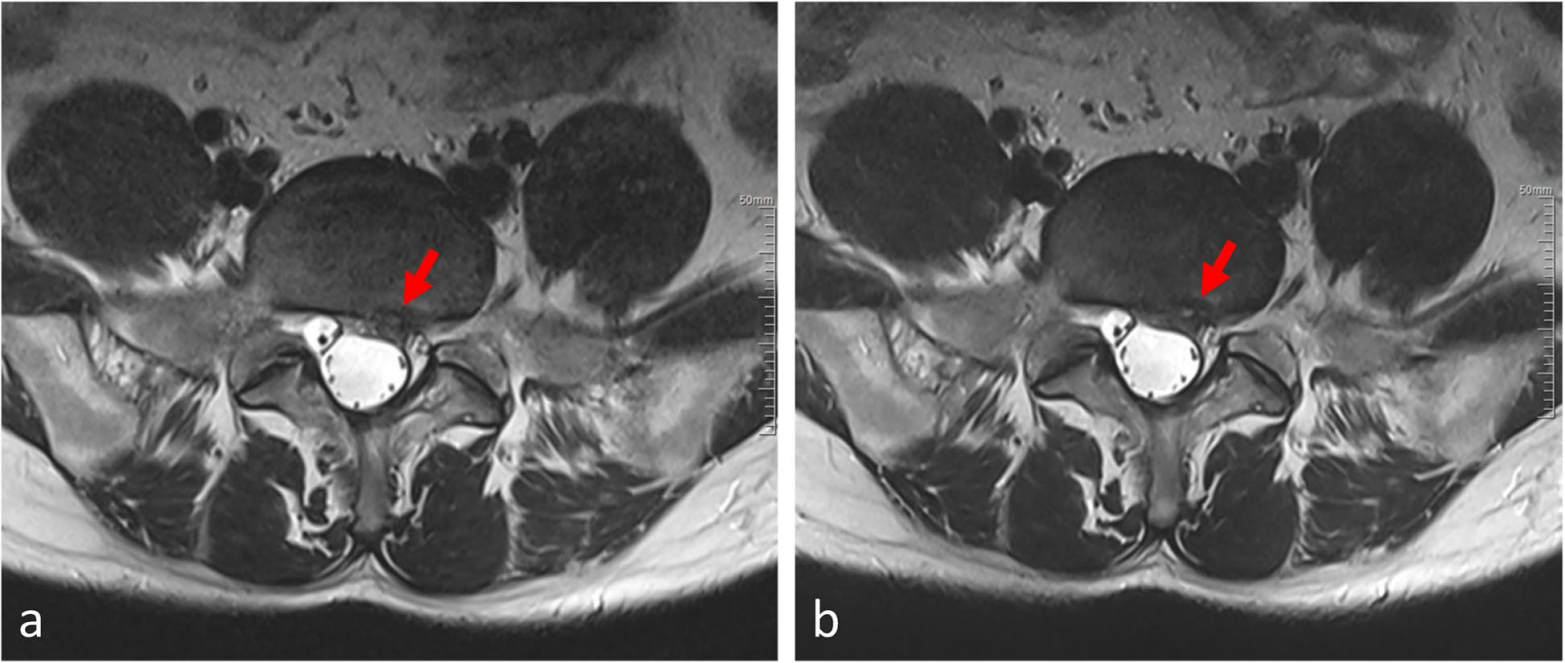
A 38-year-old male with left leg pain. Both TSE-SD (**a**) and TSE-DL (**b**) acquisitions of transverse T2-weighted imaging show the lumbar disc protruding posteriorly to the left (arrows). TSE-SD, Standard turbo spin-echo; TSE-DL, Turbo spin-echo with deep learning reconstruction

**Fig. 4:**
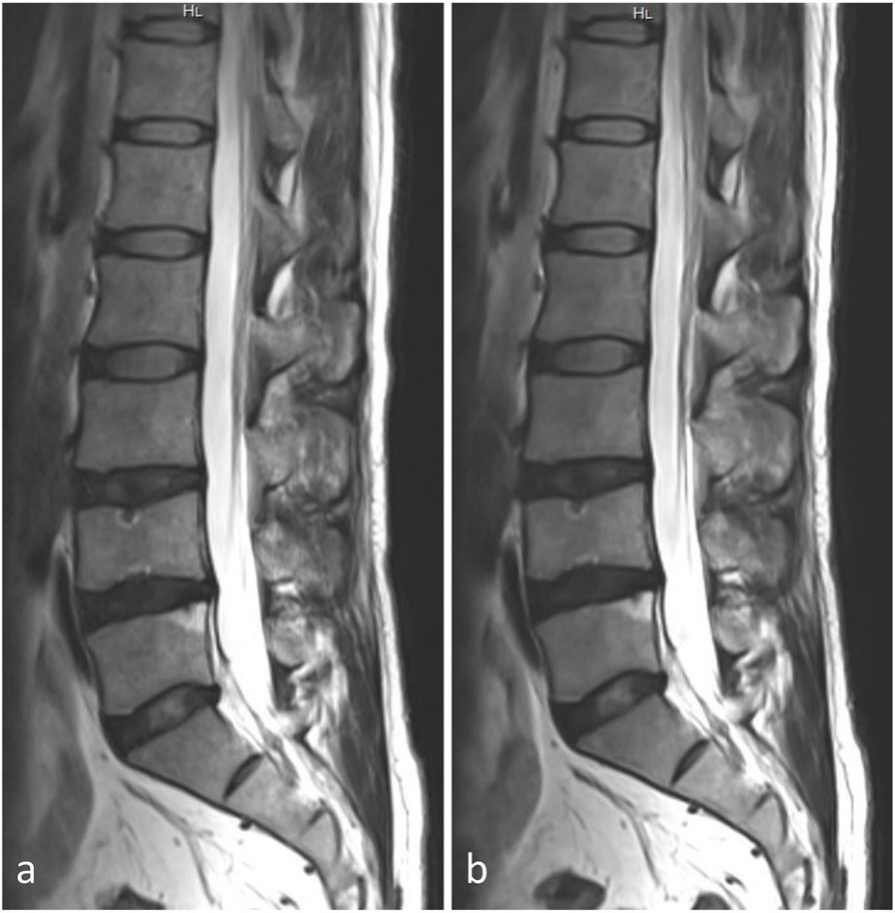
A 36-year-old male presented with low back pain. Sagittal planes are T2-weighted images, with TSE-SD scanning images on the left (**a**) and images obtained with TSE-DL on the right (**b**). Both images show the Schmorl nodes at the L4–L5 vertebrae. TSE-SD, Standard turbo spin-echo; TSE-DL, Turbo spin-echo with deep learning reconstruction

Detection rates of major pathologies by TSE-SD and TSE-DL are shown in Table 5, with no evidence of significantly higher detection rates by TSE-SD compared with TSE-DL (*p* ≥ 0.219). Figures 3 and 4 show cases with lumbar disc herniation and Schmorl nodes, respectively, both of which were well represented and could be diagnosed on TSE-SD and TSE-DL images.

**Table 5:** Detection of common abnormities by TSE-SD and TSE-DL

	Abnormalities reported				*p-*value
	Total	With both TSE-SD and TSE-DL	With TSE-SD only	With TSE-DL only	
Spinal stenosis	24	21 (87.5)	1 (4.2)	2 (8.3)	1.000
Foraminal stenosis	33	27 (81.8)	3 (9.1)	3 (9.1)	1.000
Intervertebral disc degeneration	83	76 (91.6)	4 (4.8)	3 (3.6)	1.000
Disc bulge	53	47 (88.7)	5 (9.4)	1 (1.9)	0.219
Disc herniation	45	39 (86.7)	1 (2.2)	5 (11.1)	0.219
Facet synovial cyst	2	2 (100)	0 (0)	0 (0)	1.000
Modic changes	20	16 (80.0)	1 (5.0)	3 (15.0)	0.625
Schmorl nodes	11	10 (90.9)	1 (9.1)	0 (0)	1.000

Data in parentheses are percentages

*TSE-SD* Standard turbo spin-echo, *TSE-DL* Turbo spin-echo with deep learning reconstruction

## Discussion

We prospectively investigated the application of DL reconstruction in lumbar spine MRI scanning and evaluated the difference between TSE-SD and TSE-DL in terms of image quality and detection performance of common degenerative pathologies. Our results showed that the application of TSE-DL with an approximate 45% reduction in scan time could improve or at least maintain the overall image quality and was clinically feasible for the detection of common degenerative pathologies in lumbar spine MRI. Compared with TSE-SD, TSE-DL had higher SNR and was not significantly different in diagnostic confidence. With regard to the detection of common degenerative pathologies, TSE-DL showed substantial to almost perfect interreader and interprotocol reproducibility and exhibited detection rates comparable to TSE-SD.

DL reconstruction for MRI has recently gained increasing attention and shown promising results in providing a balance between scan efficiency and image quality. Sebastain et al. [16] decreased about 60% scan time by reducing the number of acquisitions in three-plane T2-weighted TSE imaging in the prostate and reported improved image quality for DL reconstruction. Lee et al. [17] found that fourfold parallel accelerated fat-suppressed T2-weighted TSE MRI with DL reconstruction had comparable subject image quality compared with twofold parallel accelerated MRI without DL reconstruction in the knee, with a scan time reduction of 47%. However, previous studies have been conducted on a limited number of sequences, whereas in clinical settings, the MRI protocol usually consists of multiple sequences. In this study, the TSE-DL protocol used for lumbar spine MRI included sagittal T1-weighted and T2-weighted imaging and transverse T2-weighted imaging with an acceleration factor of 2, and sagittal fat-suppressed T2-weighted imaging with a number of excitations of only 1, while other parameters remained consistent with the TSE-SD protocol.

The total scan time with TSE-DL was 2:55 min:s, enabling a 45% reduction compared to TSE-SD. Reducing scan time not only alleviates patient discomfort during prolonged examinations but also enables more efficient resource allocation, ultimately leading to improved patient throughput.

According to the subjective evaluation of two readers, the noise of TSE-DL was lower than that of TSE-SD. Measurement of image SNR by two readers also indicated that TSE-DL had improved SNR compared with TSE-SD. These results are consistent with previous studies [13, 18, 19]. For instance, Bash et al. [13] enrolled 61 patients undergoing lumbar spine MRI and found that the SNR of fast DL imaging sequences was better than that of standard sequences. We also found that TSE-DL was comparable to TSE-SD in sharpness of anatomic structures. Similar results were reported by Yasaka et al. [20], who showed better results for DL-reconstructed cervical spine sagittal T2-weighted MRI compared with standard MRI for the depiction of anatomic structures except for disc and foramina by one reader. In terms of artifacts, there was no evidence of a significant difference in non-fat-suppressed sequences between the two protocols, while TSE-DL obtained a lower score than TSE-SD in sagittal fat-suppressed T2-weighted images. The common artifacts seen on TSE-DL images were residual aliasing artifacts, appearing as ghosts inside or outside the object of interest [21]. Almansour et al. [5] found that the residual aliasing artifact was one of the main sources of artifacts for spine MRI due to undersampling associated with accelerated acquisition, similar to our observations. As a result, the subjective artifact score of TSE-DL was lower than that of TSE-SD for sagittal fat-suppressed T2-weighted images. Nevertheless, diagnostic confidence of TSE-DL did not appear to be much influenced by the low artifact score, with no evidence of a significant difference with TSE-SD. Last, TSE-DL yielded a comparable overall image quality to TSE-SD, consistent with previous studies [22, 23]. Our results indicated that DL reconstruction for the whole lumbar spine MRI protocol at a scan time reduction of 45% would not reduce the overall image quality. Regarding the detection performance of common degenerative pathologies, we observed for TSE-DL a similar detection rate to TSE-SD. Interprotocol and interreader agreement were substantial to almost perfect. These results are in line with previous studies on spine MRI [5, 6] and hand and wrist MRI [24].

There are several limitations in our study. First, this experiment was done on a single MRI scanner in a single center with a small sample size. Although some statistical significance was achieved, it is necessary to expand the sample size and try it on other scanners and field strengths to observe whether the same results can be obtained. Second, no preliminary calculation of the sample size was done, so that no distinction among primary, secondary, and exploratory endpoints was defined, and the correction of the *p*-value threshold for statistical significance was done a posteriori. Third, only two radiologists participated in the reading sessions. More radiologists with different experience levels are also needed to generalize our results. Fourth, only a limited number of degenerative disorders were included, and patients with a history of lumbar spine surgery or metal implants were excluded. Some other lumbar spine disorders such as vertebral hemangiomas, vertebral tumors, masses inside or compressing the spinal cord, and other complex conditions, as well as post-surgery and post-implant conditions, should be studied to evaluate the utility of DL reconstruction in the real clinical setting. Fifth, the data analysis conducted in this study was limited to the evaluation of image quality, interreader or interprotocol agreement, and detection rate of common pathologies. However, no noninferiority or equivalence statistical testing was performed between TSE-DL and TSE-SD in terms of diagnostic test comparison. Therefore, our findings should be interpreted with caution. Finally, this experiment was conducted on non-contrast-enhanced lumbar spine MRI sequences. Whether the contrast agent would affect the measurement results still remains unclear and warrants further studies.

In conclusion, the TSE-DL protocol with a 45% reduction in scan time showed similar overall image quality in lumbar spine MRI when compared to TSE-SD for degenerative lumbar spine diseases. Application of TSE-DL is clinically feasible for detecting common degenerative abnormalities, with comparable diagnostic confidence and detection rate to TSE-SD. Future studies are warranted to determine the diagnostic equivalence between TSE-DL and TSE-SD with a large number of participants and readers. Moreover, the utility of DL reconstruction of lumbar spine MRI in a real clinical situation with other types of abnormities, various patient conditions, and potential application of contrast medium should be studied.

### Supplementary Information

**Supplementary Material 1.**

## Data Availability

The datasets used or analyzed during the current study are available from the corresponding author upon reasonable request.

## Abbreviations

DL: Deep learning
MRI: Magnetic resonance imaging
SNR: Signal-to-noise ratio
TSE-DL: Turbo spin-echo with DL reconstruction
TSE-SD: Standard turbo spin-echo
