# Supplementary material for: Deep learning reconstruction for lumbar spine MRI acceleration: a prospective study
Source: Eur Radiol Exp. 2024 Jun 21;8:67. doi: 10.1186/s41747-024-00470-0 (PMC11189847; doi:10.1186/s41747-024-00470-0)
Supplement: Supplementary file 1 — Supplementary Material 1. [file 41747_2024_470_MOESM1_ESM.pdf]

Deep learning reconstruction for lumbar spine MRI acceleration: a prospective study

ELECTRONIC SUPPLEMENTARY MATERIAL

Appendix 1: Details on the dedicated software and the deep learning algorithm used

SubtleMR is an FDA-cleared and CE-marked software which constructs a deep learning model based on the Deep Back-Projection Network (DBPN) [1] to improve the quality of input images. DBPN employs multiple projection blocks stacked together to achieve layer-by-layer reconstruction of images. Within each projection block, multiple convolutional and deconvolutional operations simulate the traditional back-projection process to enhance image details and clarity. The dense connections between back-projection blocks help alleviate the vanishing gradient problem, facilitating better learning of image details and structural information by the network. SubtleMR is trained and validated on a dataset comprising over a million pairs of low- and high-quality MR images. This dataset is diverse and encompasses images from various vendors (such as GE, Philips, Siemens, Hitachi), scanner models, field strengths, and clinical sites. It covers a wide range of tissue contrasts, acquisition parameters, patient anatomies, and varying image qualities [2]. The diversity and heterogeneity of samples contribute to the robustness and generalization capabilities of the model. The model is designed for MRI imaging across various modalities, including T1 weighted (T1), T2 weighted (T2), proton density weighted (PD), short tau inversion recovery (STIR), fluid-attenuated inversion recovery (FLAIR), double inversion recovery (DIR), diffusion weighted (DWI), perfusion weighted (PWI), magnetic resonance angiography (MRA) and venography. It is compatible with MRI machines from major vendors such as GE, Philips, Siemens, and Hitachi. Additionally, it is tailored for a wide range of anatomies, including ankle, brain, elbow, foot, hand, hip, knee, prostate, shoulder, spine, upper leg, and wrist.

References:

[1] Haris M, Shakhnarovich G, Ukita N. Deep back-projection networks for super-resolution. In Proceedings of the IEEE conference on computer vision and pattern recognition. 2018: 1664-1673.

[2] Bash S, Johnson B, Gibbs W, et al. Deep learning image processing enables 40% faster spinal MR scans which match or exceed quality of standard of care: a prospective multicenter multireader study. Clinical Neuroradiology, 2022, 32(1): 197-203.

Table S1. Subjective evaluation of sagittal T2-weighted imaging by two readers.

	Reader	TSE-SD	TSE-DL	*p* value	Cohen's kappa
Sharpness	1	3.84±0.37	4.00	0.020	0.45 (0.28,0.63)
	2	3.97±0.18	3.97±0.18	1.000	
Artifacts	1	3.97±0.18	3.94±0.25	0.322	0.13 (-0.07,0.32)
	2	3.97±0.18	3.90±0.30	0.322	
Noise	1	3.84±0.37	4.00	0.021	0.69 (0.54,0.85)
	2	3.84±0.37	4.00	0.021	
Overall image quality	1	3.94±0.25	4.00	0.164	0.61 (0.44,0.77)
	2	3.90±0.30	3.94±0.25	0.565	
Diagnostic confidence	1	4.00	4.00	1.000	0.44 (0.24,0.63)
	2	4.00	4.00	1.000	

Data in parentheses are 95% confidence intervals. Image quality was evaluated with use of a four-point Likert scale. For sharpness, overall image quality and diagnostic confidence, the scoring system was: 1, poor; 2, fair; 3, good; and 4, excellent. For artifacts and noise, the scoring system was: 1, severe; 2, moderate; 3, mild; and 4, none. *TSE-SD* Standard turbo spin-echo, *TSE-DL* Turbo spin-echo with deep learning reconstruction.

Table S2. Subjective evaluation of sagittal fat-suppressed T2-weighted imaging by two readers.

	Reader	TSE-SD	TSE-DL	*p* value	Cohen's kappa
Sharpness	1	3.84±0.37	3.84±0.37	1.000	0.65 (0.52,0.78)
	2	3.87±0.34	3.74±0.45	0.101	
Artifacts	1	3.97±0.18	3.26±0.45	<0.001	0.53 (0.39,0.68)
	2	3.74±0.45	3.23±0.67	0.001	
Noise	1	3.74±0.45	3.94±0.25	0.014	0.66 (0.53,0.79)
	2	3.68±0.48	3.84±0.37	0.025	
Overall image quality	1	3.90±0.30	3.84±0.37	0.412	0.68 (0.55,0.81)
	2	3.74±0.45	3.74±0.46	1.000	
Diagnostic confidence	1	3.94±0.25	3.81±0.40	0.101	0.59 (0.44,0.75)
	2	3.84±0.37	3.84±0.37	1.000	

Data in parentheses are 95% confidence intervals. Image quality was evaluated with use of a four-point Likert scale. For sharpness, overall image quality and diagnostic confidence, the scoring system was: 1, poor; 2, fair; 3, good; and 4, excellent. For artifacts and noise, the scoring system was: 1, severe; 2, moderate; 3, mild; and 4, none. *TSE-SD* Standard turbo spin-echo, *TSE-DL* Turbo spin-echo with deep learning reconstruction.

Table S3. Subjective evaluation of transverse T2-weighted imaging by two readers.

	Reader	TSE-SD	TSE-DL	*p* value	Cohen's kappa
Sharpness	1	3.71±0.46	3.94±0.25	0.022	0.58 (0.44, 0.72)
	2	3.87±0.34	3.94±0.25	0.414	
Artifacts	1	3.90±0.30	3.97±0.18	0.163	0.36 (0.17,0.54)
	2	3.84±0.45	3.84±0.37	1.000	
Noise	1	3.39±0.50	3.97±0.18	<0.001	0.53 (0.42,0.64)
	2	3.71±0.46	3.94±0.25	0.044	
Overall image quality	1	3.74±0.44	3.97±0.18	0.022	0.67 (0.55,0.79)
	2	3.77±0.50	3.94±0.25	0.131	
Diagnostic confidence	1	3.90±0.30	4.00	0.085	0.62 (0.46,0.79)
	2	3.87±0.34	3.97±0.18	0.187	

Data in parentheses are 95% confidence intervals. Image quality was evaluated with use of a four-point Likert scale. For sharpness, overall image quality and diagnostic confidence, the scoring system was: 1, poor; 2, fair; 3, good; and 4, excellent. For artifacts and noise, the scoring system was: 1, severe; 2, moderate; 3, mild; and 4, none. *TSE-SD* Standard turbo spin-echo, *TSE-DL* Turbo spin-echo with deep learning reconstruction.

Table S4. Signal-to-noise ratio measurement of sagittal T2-weighted imaging by two readers.

		Reader	TSE-SD	TSE-DL	*p* value	ICC
Vertebra	L1	1	146.4±37.2	205.9±54.5	<0.001	0.83 (0.73,0.90)
		2	135.3±40.5	195.9±59.6	<0.001	
	L2	1	145.4±38.4	203.3±59.3	<0.001	0.86 (0.76,0.91)
		2	134.0±41.1	192.9±61.8	<0.001	
	L3	1	136.5±36.1	193.3±58.5	<0.001	0.86 (0.78,0.92)
		2	127.2±40.0	184.1±82.8	<0.001	
	L4	1	132.5±36.2	186.2±56.2	<0.001	0.84 (0.72,0.90)
		2	121.0±35.8	174.8±55.9	<0.001	
	L5	1	138.9±40.8	194.3±60.7	<0.001	0.83 (0.70,0.90)
		2	125.9±37.7	181.8±55.4	<0.001	
Intervertebral disc	L1/L2	1	86.7±43.4	127.3±63.4	<0.001	0.93 (0.88,0.95)
		2	81.1±41.6	122.9±66.9	<0.001	
	L2/L3	1	74.6±40.2	112.3±63.2	<0.001	0.94 (0.91,0.96)
		2	72.0±40.2	109.1±65.2	<0.001	
	L3/L4	1	63.8±35.4	96.7±57.5	<0.001	0.93 (0.89,0.96)
		2	61.0±37.3	93.0±59.8	<0.001	
	L4/L5	1	51.2±28.0	73.8±42.6	<0.001	0.87 (0.80,0.92)
		2	47.1±29.0	72.5±44.9	<0.001	
	L5/S1	1	69.9±36.8	103.3±58.0	<0.001	0.87 (0.78,0.92)
		2	63.2±33.3	92.9±53.5	<0.001	

Muscle	1	60.2±16.5	84.4±24.7	<0.001	0.79 (0.68,0.87)
	2	56.4±18.6	85.3±33.9	<0.001	
Cerebrospinal fluid	1	310.5±101.1	447.3±147.7	<0.001	0.86 (0.75,0.91)
	2	280.9±88.2	420.4 ± 139.0	<0.001	
Fat	1	376.1±93.8	535.9±151.3	<0.001	0.67 (0.50,0.78)
	2	348.3 ± 116.3	513.4 ± 177.5	<0.001	

Data in parentheses are 95% confidence intervals. *ICC* Intraclass correlation coefficient, *TSE-SD* Standard turbo spin-echo, *TSE-DL* Turbo spin-echo with deep learning reconstruction.

Tabel S5. Signal-to-noise ratio measurement of sagittal fat-suppressed T2-weighted imaging by two readers.

		Reader	TSE-SD	TSE-DL	*p* value	ICC
Vertebra	L1	1	100.8±46.0	124.5±59.2	<0.001	0.86 (0.77,0.91)
		2	95.9±55.7	112.8±49.8	0.006	
	L2	1	93.0±61.2	109.4±56.2	0.015	0.77 (0.64,0.86)
		2	81.5±53.1	96.4±46.4	0.006	
	L3	1	74.7±38.5	95.3±49.2	<0.001	0.89 (0.81,0.93)
		2	69.2±43.7	85.5±42.2	<0.001	
	L4	1	69.7±34.6	91.8±43.8	<0.001	0.90 (0.82,0.94)
		2	64.4±34.7	84±37.6	<0.001	
	L5	1	72.9±33.1	91.7±41.4	<0.001	0.86 (0.77,0.92)
		2	68.4±35.8	82.7±34.2	0.001	
Intervertebral disc	L1/L2	1	284.5±144.5	329.1±153.9	<0.001	0.83 (0.74,0.89)
		2	274.0±142.2	302.9±128.2	0.039	
	L2/L3	1	238.8±123.6	287.4±143.6	<0.001	0.88 (0.80,0.92)
		2	235.3±124.4	266.0±124.6	0.005	
	L3/L4	1	196.1±107.5	244.6±138.7	<0.001	0.90 (0.84,0.94)
		2	190.0±108.0	224.1±119.9	<0.001	
	L4/L5	1	160.1±96.3	191.6±117.9	0.001	0.90 (0.82,0.94)
		2	147.0±84.6	171.5±100.2	0.005	
	L5/S1	1	227.1±120.0	268.1±129.8	<0.001	0.89 (0.83,0.93)
		2	217.3±137.7	249.0±134.0	0.011	
Muscle		1	114.8±30.2	136.0±37.1	<0.001	0.58 (0.39,0.73)
		2	109.5±38.5	122.3±24.6	0.023	
Cerebrospinal fluid		1	1015.4±346.0	1178.9±337.9	<0.001	0.65 (0.44,0.78)
		2	927.0±331.9	1044.3 ± 271.2	0.005	
Fat		1	53.9±40.0	67.5±53.6	0.001	0.54

	2	45.8±61.5	54.7±45.3	0.048	(0.34,0.69)

Data in parentheses are 95% confidence intervals. *ICC* Intraclass correlation coefficient, *TSE-SD* Standard turbo spin-echo, *TSE-DL* Turbo spin-echo with deep learning reconstruction.

Table S6. Signal-to-noise ratio measurement of transverse T2-weighted imaging by two readers.

		Reader	TSE-SD	TSE-DL	*p* value	ICC
Nerve root	Left	1	68.5±23.7	117.0±33.5	<0.001	0.75 (0.62,0.84)
		2	68.9±25.2	110.4±42.5	<0.001	
	Right	1	61.6±28.2	106.6±38.0	<0.001	0.63 (0.45,0.76)
		2	65.5±25.8	102.7±42.2	<0.001	
Psoas major muscle	Left	1	26.7±9.1	45.8±12.9	<0.001	0.79 (0.62,0.88)
		2	24.9±8.2	39.8±12.5	<0.001	
	Right	1	20.7±5.9	36.3±9.8	<0.001	0.73(0.57,0.83)
		2	19.5±5.8	31.8±10.3	<0.001	
Intervertebral disc		1	44.2±18.2	75.9±27.8	<0.001	0.82 (0.72,0.89)
		2	41.4±19.1	69.4±30.3	<0.001	
Cerebrospinal fluid		1	165.5±51.7	276.7±64.4	<0.001	0.77 (0.65,0.85)
		2	163.1 ± 51.0	259.5±75.6	<0.001	
Fat		1	212.9±60.8	353.8±82.5	<0.001	0.76 (0.60,0.86)
		2	195.4 ± 61.0	313.0 ± 117.1	<0.001	

Data in parentheses are 95% confidence intervals. *ICC* Intraclass correlation coefficient, *TSE-SD* Standard turbo spin-echo, *TSE-DL* Turbo spin-echo with deep learning reconstruction.

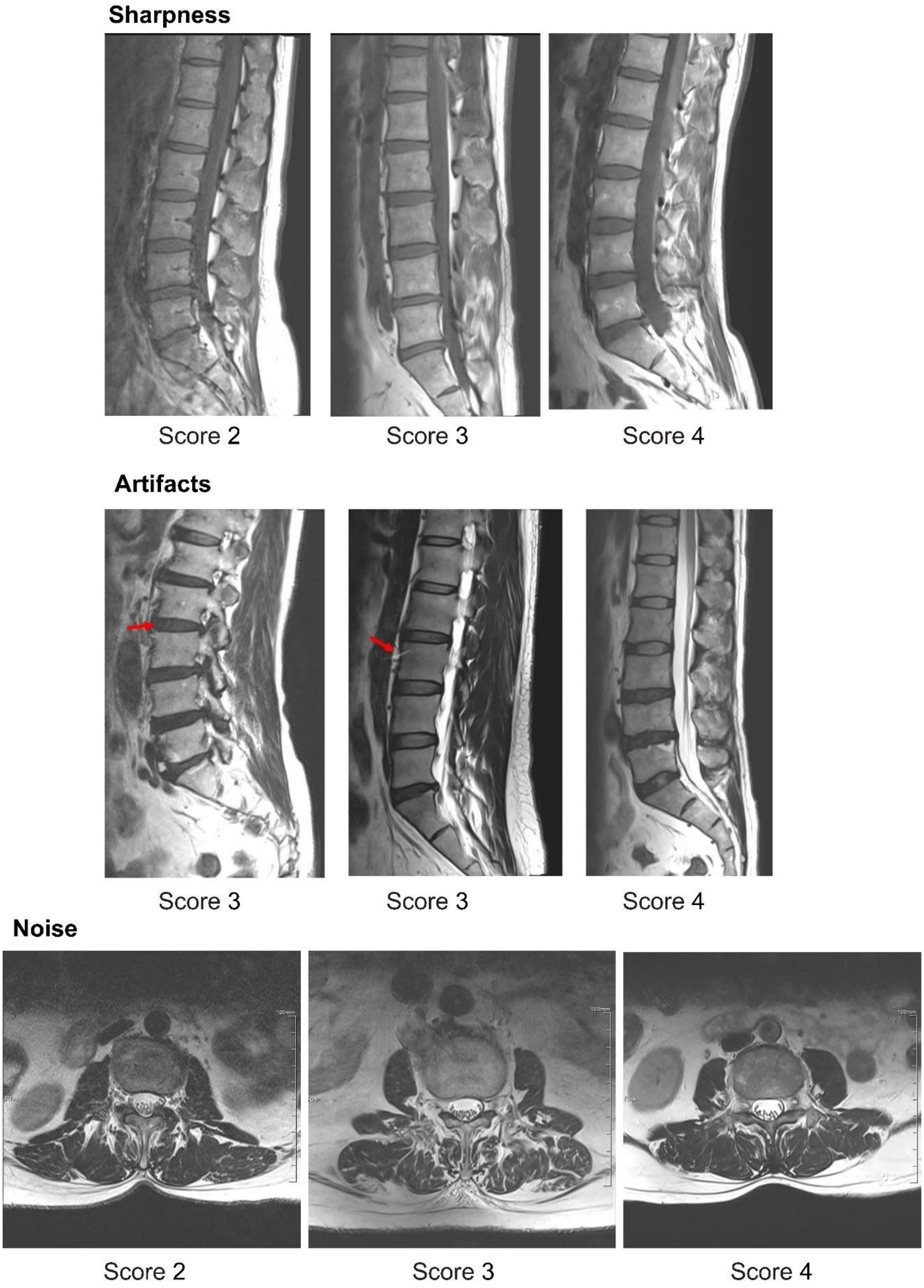
Sharpness
Score 2
Score 3
Score 4
Artifacts
Score 3
Score 3
Score 4
Noise
Score 2
Score 3
Score 4

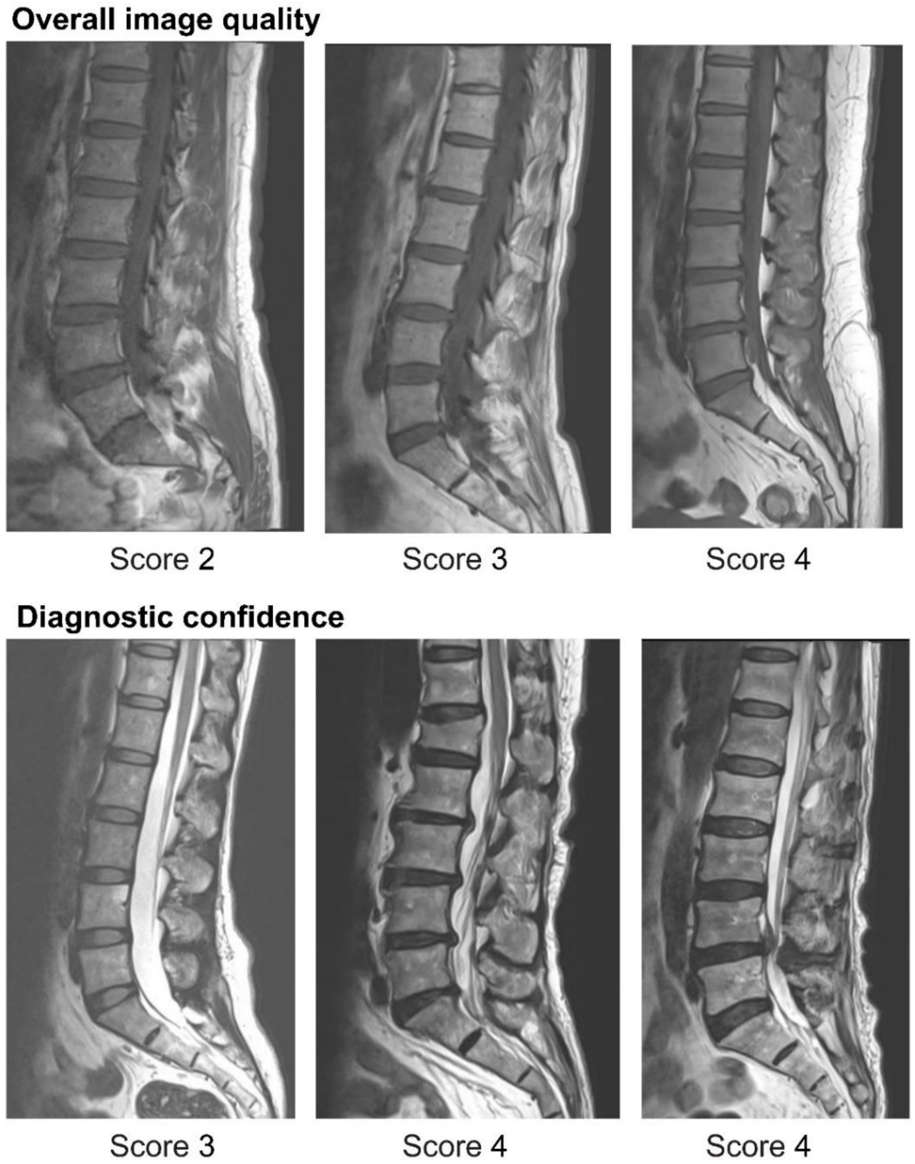

Figure S1. Examples of the application of the 4-point Likert scale to lumbar spine MRI scans to evaluate sharpness of anatomic structures, artifacts, noise, overall image quality and diagnostic confidence. Note that for certain criteria, images received scores of 1 or 2 were lacking.

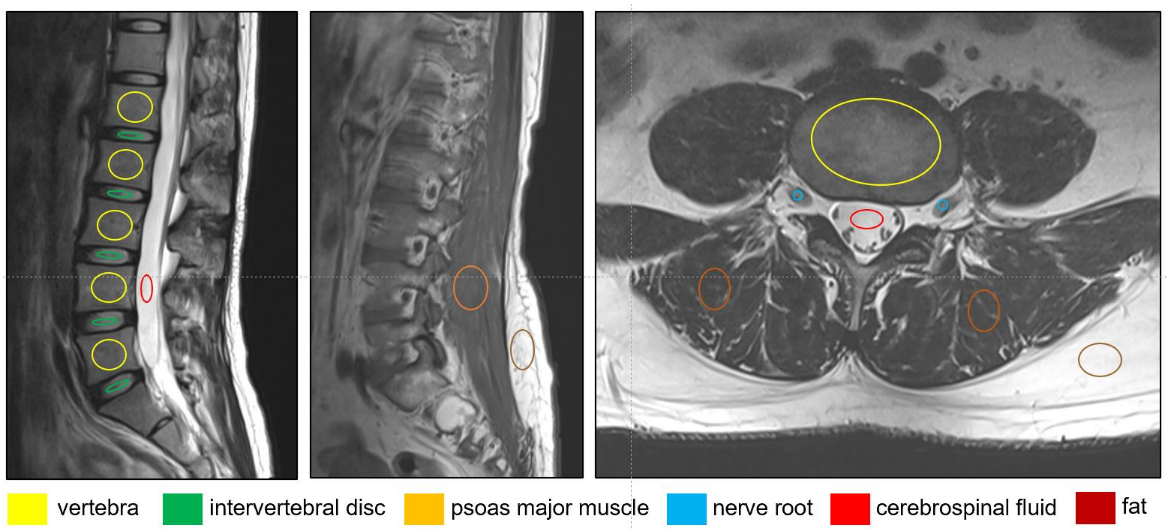

Figure S2. Region of interest placement on sagittal and transverse images for measurement of signal-to-noise ratio.
